# The unique role of cardiovascular magnetic resonance imaging in acute myocarditis

**DOI:** 10.12688/f1000research.14857.1

**Published:** 2018-07-30

**Authors:** Michael Chetrit, Matthias G. Friedrich

**Affiliations:** 1 Department of Medicine, McGill University, Montreal, QC, Canada; 2 Department of Diagnostic Radiology, McGill University, Montreal, QC, Canada; 3 Department of Medicine, Heidelberg University, Heidelberg, Germany

**Keywords:** Cardiovascular Magnetic Resonance, Acute Myocarditis

## Abstract

This article addresses the specific diagnostic information provided by cardiovascular magnetic resonance (CMR) in patients with suspected acute myocarditis. It gives an overview of the current evidence of the ability of CMR to detect myocardial inflammation and discusses the added value as well as its limitations in clinical settings. Because of the large variety of symptoms and the limited specificity of other non-invasive procedures, the identification of myocardial inflammation is of paramount importance. Because of its accuracy in imaging ventricular volumes and function and its unique ability to visualize myocardial edema, scar, and other tissue abnormalities, CMR has emerged as the prime non-invasive diagnostic tool in patients with acute myocarditis. The presence of myocardial inflammation is not specific to viral myocarditis or other forms of acute myocardial injury, and the regional distribution within the myocardium helps differentiate acute myocarditis from other diseases. The currently recommended diagnostic criteria (Lake Louise Criteria) include markers for hyperemia/capillary leak, edema, and inflammatory scarring. Their diagnostic accuracy of close to 80% is satisfactory to rule in myocarditis, yet the negative predictive value is less than 70%. Novel CMR techniques, especially T1 and T2 mapping, have been shown to further improve the diagnostic utility.

## Introduction

Acute myocarditis is typically understood as an acute inflammation caused by a viral infection, although inflammation per se is a non-specific response to many stressors such as toxins (for example, chemotherapy), autoimmune disease, infarction, or a catecholamine ^1^. Viral myocarditis is a frequent complication of systemic viral infection. More than 50% of patients with influenza, for example, have electrocardiography (ECG) changes indicating myocardial involvement ^2^. Because patients with myocarditis can present with a wide range of clinical patterns such as fatigue, palpitations, or just lack of physical fitness, the advent of cardiovascular magnetic resonance (CMR) has improved the detection of myocardial involvement and thereby clinicians’ awareness of this disease. Although there is no established specific treatment for viral myocarditis, ruling it in or out has important clinical implications, such as avoiding other diagnostic tests, especially coronary angiography to rule out coronary artery disease, or advising the patient to withhold extreme physical stress for 3 to 4 weeks. Other diagnostic tools for acute myocarditis have significant limitations. The ECG is notoriously non-specific and (because of the frequent inferolateral location of myocarditis) not sensitive either ^3^. Many patients with acute myocarditis have normal left ventricular function; thus, an echocardiogram not only lacks specificity for viral infection but also is hampered by a limited sensitivity. Cardiac troponins are very sensitive to myocardial injury yet are not specific for inflammation and lack information on the location. Endomyocardial biopsy allows a definitive diagnosis but is not very sensitive and is highly invasive and costly. Current guidelines promote its use only in patients with an acute unexplained heart failure complicated by hemodynamic instability ^4^. Given the clinical ambiguity surrounding myocarditis and the pertinence of establishing a diagnosis, a reliable non-invasive tool able to characterize the properties of the myocardium such as CMR has emerged as a useful tool.

## Diagnostic challenges of acute myocarditis

In 1995, the World Health Organization in concert with the International Society and Federation of Cardiology defined myocarditis as an inflammatory disease of the heart by using immunological and histological criteria with a focus on the presence of myocardial inflammation. The clinical diagnosis of myocarditis, however, remains a challenge because of the wide spectrum of symptoms and disease states. The recommended European Society of Cardiology criteria for the clinical diagnosis of myocarditis are based on new-onset dyspnea, palpitations, or chest discomfort plus evidence of myocardial damage in the absence of a severely stenosed coronary artery. These criteria, however, were lacking proof of the presence and location of myocardial inflammation. Biomarkers of inflammation such as C-reactive protein and erythrocyte sedimentation rate have been considered but are non-specific and can be normal on presentation, limiting their diagnostic contribution.

## The role of cardiovascular magnetic resonance

Magnetic resonance imaging (MRI) has propelled itself to the forefront of cardiac imaging because of its spatial resolution, quantitative accuracy, and inter-observer consistency. Unique to this modality is its ability to provide information outside of size and function, particularly in characterizing tissue abnormalities, especially in non-ischemic cardiomyopathies ^5^. MRI is based on the absorption and emission of energy from the nuclei of atoms, protons in particular, when placed in a magnetic field ^6^. It is free of known significant side effects and does not require radioactivity. Hydrogen atoms exist ubiquitously in the human body, especially including free water (such as in edema), making MRI a powerful imaging modality for identifying regional inflammation. Furthermore, contrast-enhanced CMR can visualize irreversible injury (necrosis and scar) ^7^. The most widely used technique, late gadolinium enhancement (LGE), has been validated against biopsy-proven scar, and findings of areas with increased signal after gadolinium administration have important prognostic implications in various cardiac pathologies.

In 2009, the Lake Louise Consensus Group recommended a standard protocol that would best identify myocardial inflammation by using CMR ^8^. Diagnostic targets for the three recommended CMR criteria were edema, hyperemia, capillary leak, and necrosis. Two out of three “Lake Louise Criteria” allow for a correct diagnosis of acute myocarditis in about 80% of cases ^8, 9^.

The Lake Louise Criteria were quickly adopted and their performance was tested in a broader population. Biesbroek *et al*. verified the additional value of CMR to the original clinical criteria proposed in 2013 ^10^. CMR was able to confirm the diagnosis of acute myocarditis in the majority of patients with a clinical suspicion of acute myocarditis based on the clinical criteria. In the group with insufficient clinical criteria, almost half underwent CMR as a primary investigation and were provided with a diagnosis and thus spared an invasive coronary angiogram, endomyocardial biopsy, or inappropriate initiation of therapy. Moreover, amongst those with a clinical presentation suggestive of acute myocarditis based on the aforementioned criteria, 18% were provided an alternative diagnosis based on the CMR findings, the most prevalent of which was myocardial infarction. This has important clinical implications because the therapeutic plan is different and medications to prolong survival are well established in coronary disease.

These larger studies were also able to identify some of the limitations of the Lake Louise Criteria, namely the inability to detect myocardial inflammation in various myocarditis subtypes as well as the low performance of these criteria in the chronic stages of myocarditis ^9^. This is of concern given that the persistence of myocardial inflammation in the chronic phase is currently believed to be a key factor in the progression to a dilated cardiomyopathy and would be an area for potential intervention. Overall, clinical centers have encountered varying success rates, and sensitivities range from 60% to 85% and specificities range from 68% to 100% ^11^. Whereas the diagnostic accuracy is close to 80%, which is satisfactory to rule in myocarditis, the negative predictive value of 70% leaves room for improvement.

## Novel cardiovascular magnetic resonance techniques

**Figure 1.**
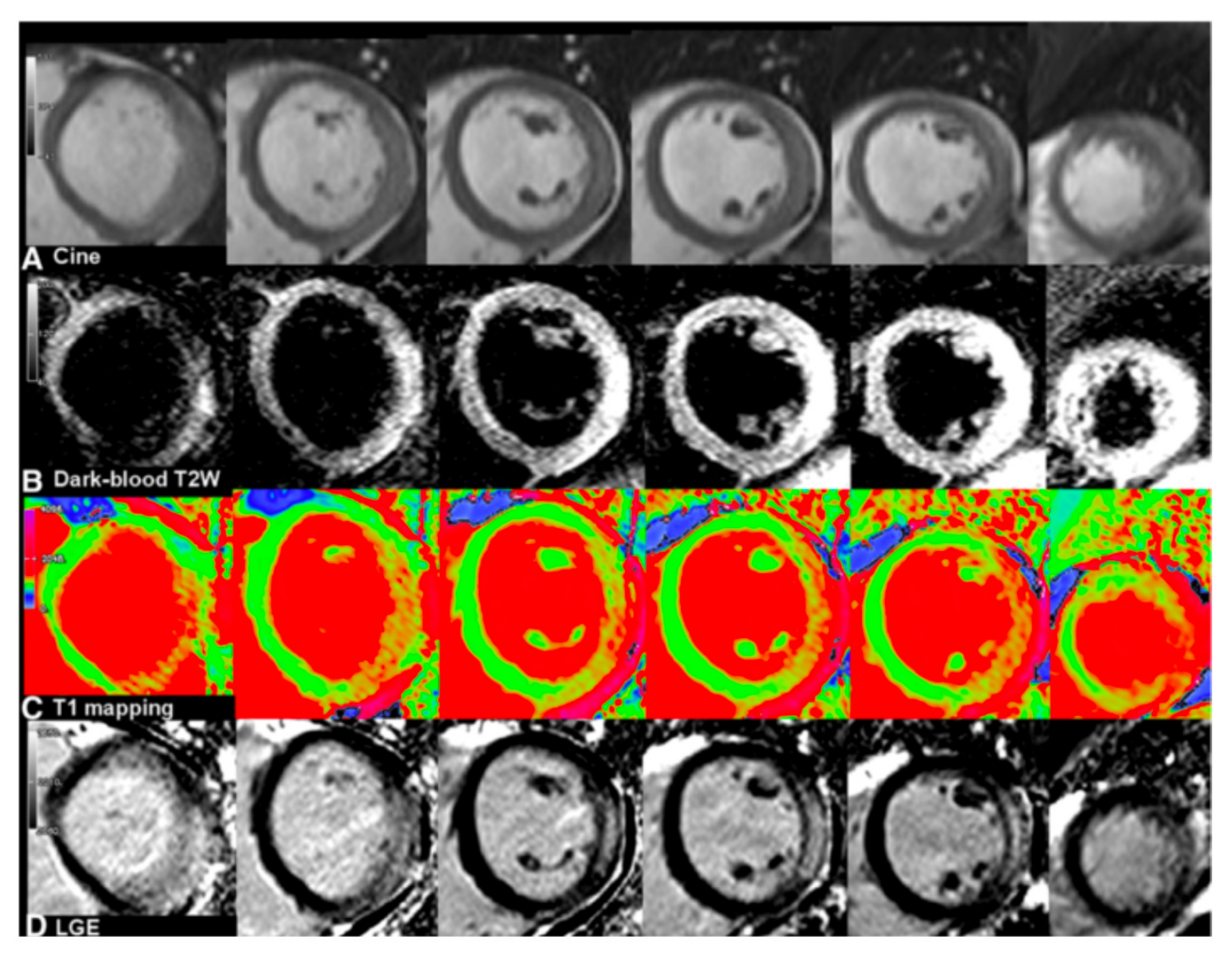
Cardiovascular magnetic resonance findings in a patient with acute myocarditis (short-axis stack). ( **Row A**) Systolic cine images. ( **Row B**) Dark-blood, T2-weighted images indicating edema. ( **Row C**) T1 maps with red indicating increased values. ( **Row D**) Late gadolinium-enhanced (LGE) images indicating irreversible injury. Reprinted with permission from BioMed Central Ltd ^19^.

**Figure 2.**
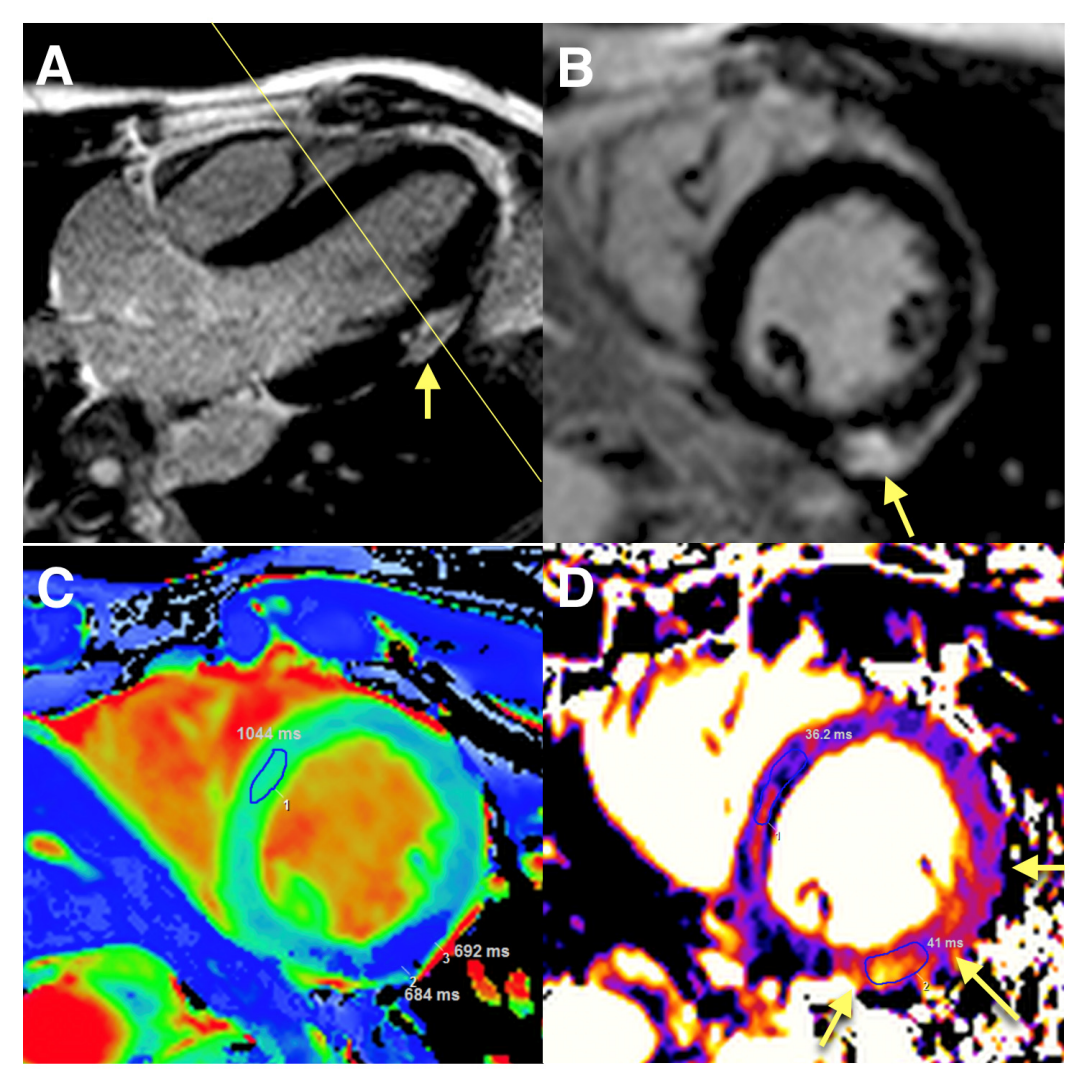
Cardiovascular magnetic resonance findings in a patient with acute myocarditis (short-axis stack). Cardiovascular magnetic resonance findings suggestive of acute myocarditis. ( **A**) Late gadolinium-enhanced long-axis view with a subepicardial lesion in the mid-ventricular segment (arrow). ( **B**) Short-axis view of the same lesion (arrow). ( **C**) Post-contrast T1 map with marked T1 reduction in the same region. ( **D**) Short-axis T2 map with an increased T2, indicating the associated edema (arrows).

Novel techniques, notably T1 and T2 mapping, including extracellular volume (ECV) quantification, have emerged as accurate techniques in the characterization of the myocardium ( Figure 1). In patients with myocarditis, these advancements appear to overcome some of the limitations of the Lake Louise Criteria ^12^. The mapping techniques provide quantitative data on the magnetic properties of the tissue, typically referred to as the relaxation times T1 and T2 and thus are less susceptible to the limitations of the often-subjective or even just visual assessment of signal intensity. More importantly, however, inflammation appears to affect native (non-contrast) T1 and T2 strongly enough to be identified without the use of contrast agents ^13^. Combined pre- and post-contrast T1 mapping can also be used to quantify ECV in acute and chronic phases of myocarditis. Probably better than edema-sensitive (T2-weighted) CMR imaging, native myocardial T2 was shown to be sensitive in assessing myocardial inflammation and reversible injury, allowing assessment for acute/active (as opposed to healed) inflammation ^14^ ( Figure 2). T2 mapping was also recently shown to predict functional outcome ^15^. In consequence, T1 and T2 mapping can improve the overall diagnostic performance of CMR when diagnosing suspected myocarditis ^12^. Bohnen *et al*. not only concluded that native myocardial T1 and T2 are able to differentiate acute from healed myocarditis but also found that the combination of LGE images and ECV was diagnostic in both acute and chronic phases of the disease ^16^. Early experience with these techniques has demonstrated variability between scanner types and individuals, rendering validated diagnostic thresholds difficult to define ^17^. As such, harmonization of magnet strengths, mapping sequences, and mapping analysis is under way.

CMR image-derived myocardial strain analysis is another novel technique. Several strain tools are available to quantitatively assess myocardial deformation by CMR. Though not specific for inflammation, strain can provide additional information on subtle systolic or diastolic dysfunction, even before a detectable drop in ejection fraction. Strain may be particularly useful when combined with T2 mapping and LGE ^18^.

## Impact on treatment and prognosis

To date, there are no established therapies specific to the treatment of myocarditis. When faced with systolic dysfunction or in the presence of pericardial effusion, current recommendations include treating patients with the respective guideline-directed therapy for heart failure and pericarditis. The prognosis associated with an episode of acute myocarditis varies and was traditionally felt to be dependent on the clinical presentation and various measurable parameters, including New York Heart Association class and left ventricular ejection fraction as measured by echocardiography. Some more recent studies have tried to address risk stratification by using CMR. Depending on the population, the presence of LGE was associated with a more or less increased risk for future events, whereas a normal CMR study on the other hand is associated with a favorable outcome ^20, 21^.

## Conclusions

CMR provides a unique value in patients with suspected acute myocarditis. Novel techniques of CMR relaxation time mapping (T1, T2, and ECV) are expected to further improve its diagnostic accuracy. Therefore, CMR is to be considered. The ability to non-invasively identify tissue abnormalities like edema and necrosis renders it the prime diagnostic tool in the diagnostic workup of patients with suspected myocardial inflammation.

Future research should focus on using CMR criteria as endpoints in clinical trials on therapeutic management of myocarditis and explore the diagnostic value of combining CMR criteria with seromarkers such as troponin.

## Abbreviations

CMR, cardiovascular magnetic resonance; ECG, electrocardiography; ECV, extracellular volume; LGE, late gadolinium enhancement; MRI, magnetic resonance imaging.
